# Influence of Material Selection on the Mechanical Properties of 3D-Printed Tracheal Stents for Surgical Applications

**DOI:** 10.3390/polym17162223

**Published:** 2025-08-15

**Authors:** Aurora Pérez Jiménez, Carmen Sánchez González, Sandra Pérez Teresí, Noelia Landa, Cristina Díaz Jiménez, Mauro Malvé

**Affiliations:** 1 Department of Engineering, Public University of Navarra (UPNA), Campus Arrosadia, s/n, E-31006 Pamplona, Spainmauro.malve@unavarra.es (M.M.); 2 AIN—Asociacion de la Industria Navarra, Ctra. Pamplona, 1. Edif. AIN, E-31191 Cordovilla, Spain; cdiaz@ain.es; 3 Research Networking in Bioengineering, Biomaterials & Nanomedicine (CIBER-BBN), Av. Monforte de Lemos, 3-5, Pabellόn 11, Planta 0, E-28029 Madrid, Spain; 4 Instituto de Materiales Avanzados y Matemáticas (INAMAT2), Public University of Navarre (UPNA), Edificio Jerόnimo de Ayanz, Campus de Arrosadía, E-31006 Pamplona, Spain

**Keywords:** tracheal stents, biodegradable materials, radial force, mechanical properties, surgical placement

## Abstract

Endotracheal prosthesis placement is employed as a therapeutic intervention for tracheal lesions in cases where conventional surgical approaches are not feasible. The learning curve for endotracheal stent placement can vary depending on the type of stent, the training environment, and the clinician’s prior experience; however, it is generally considered moderately complex. Inadequate practice can have serious consequences, as the procedure involves a critical area such as the airway. The main risks and complications associated with inadequate technique or improper execution can include stent migration, formation of granulation tissue or hyperplasia, tracheal or pulmonary infection, obstruction or fracture of the stent, hemorrhage and tracheal perforation, among others. The purpose of the present study is to summarize important information and evaluate the role of different material features in the 3D printing manufacturing of an appropriate tracheobronchial medical device, which should be as appropriate as possible to facilitate placement during surgical practice. A complex stent design was fabricated using three different biodegradable materials, polycaprolactone (PCL), polydioxanone (PDO), and polymer blend of polylactic acid/polycaprolactone (PLA/PCL), through additive manufacturing, specifically fused filament fabrication (FFF)3D printing. Parameter optimization of the 3D printing process was required for each material to achieve an adequate geometric quality of the stent. Experimental analyses were conducted to characterize the mechanical properties of the printed stents. Flexural strength and radial compression resistance were evaluated, with particular emphasis on radial force due to its clinical relevance in preventing collapse after implantation in the trachea. The results provide valuable insights into how material selection could influence device behavior during placement to support surgical requirements.

## 1. Introduction

Tracheal stenosis represents a significant clinical challenge, especially when conventional surgical options are not viable. In this context, biodegradable tracheal stents are emerging as a promising therapeutic alternative, offering the advantage of eliminating the need for removal procedures and reducing long-term complications [1,2]. Endotracheal prostheses, or tracheal stents, are used as a therapeutic alternative in patients with pathologies that compromise the airway, such as benign or malignant tracheal stenosis, tracheal collapse, tracheomalacia, extrinsic compression by mediastinal masses, or tracheoesophageal fistulas [3,4,5]. These conditions can severely damage breathing and compromise the patient’s life, making the use of a stent an effective way to restore tracheal function in a quick and minimally invasive manner [6]. For this technique to be effective, precise anatomical placement of the stent is essential, requiring careful selection of the appropriate size, material, and geometry for each case, and employing a careful surgical technique that minimizes mechanical trauma [7,8]. The most common failures may include stent migration, granulation tissue formation, device fracture or collapse, and tracheal mucosal injuries such as perforations, necrosis, or infections [9,10,11]. These issues could be the result of poor stent selection, inadequate design, incorrect placement, or inappropriate radial force [12].

Despite the widespread use of tracheal stents in the treatment of various airway pathologies, currently available devices present significant limitations related to their materials [1,13]. Several studies have shown that the material selection of stents can directly influences the efficacy and safety of the surgical procedure during placement [6,9,14]. Therefore, characteristics such as device geometry, delivery system ergonomics, radial force, material flexibility, and adaptability to the patient’s anatomy are critical for accurate and complication-free implantation. Stents with optimized designs and controlled delivery systems have been shown to reduce the rate of technical errors, such as malposition or migration of the implant, and to facilitate safer handling during rigid bronchoscopy [15,16].

For a tracheal stent to adequately fulfill its therapeutic function, it must meet a set of minimum requirements that ensure both efficacy and clinical safety during and after the procedure. Among these requirements, the correct selection of material is fundamental, as it determines key properties such as biocompatibility, controlled degradability, and the response to the biological environment [1,13]. In addition, the device must possess appropriate mechanical properties, particularly sufficient radial force to keep the airway open without causing tissue damage, and optimal flexibility to adapt to respiratory dynamics without collapsing or generating excessive pressure points. The interaction between these factors directly affects the stent’s performance in the patient’s anatomical and functional environment, and therefore its clinical success. The selection of materials with balanced mechanical properties, particularly in terms of flexibility and radial force, has been shown to improve stent handling and intraoperative behavior [17]. Therefore, the selection of stent material should not be understood solely as a passive structural support factor, but rather as an active element that can facilitate a safer, more efficient, and more effective surgical technique [5,18].

The development of customized stents using 3D printing technologies could potentially lead to improved anatomical fit accuracy, which may help optimize postoperative performance and could simplify and shorten the surgical procedure [6,16]. Moreover, this technology appears to offer substantial versatility in material selection and combination. It may enable the use of various polymers with adjustable mechanical and degradation properties, which could be beneficial for tailoring stents to the clinical needs of individual patients [19,20,21]. The ability to print with flexible, semi-rigid, or even biodegradable polymer blends might allow for the optimization of key features such as radial strength, flexibility, and resorption time, critical factors that may contribute to effective device function and safe surgical placement. Moreover, additive manufacturing allows for the rapid and cost-effective development of prototypes for preclinical testing, thereby accelerating validation and customization processes [22,23,24].

A wide range of biodegradable materials are currently available for 3D printing, particularly compatible with techniques such as fused filament fabrication (FFF) and stereolithography (SLA). Polylactic acid (PLA) is one of the most commonly used biopolymers in this context, derived from renewable resources such as corn starch or sugarcane [25]. Its ease of printing and availability in various blends make it suitable for diverse applications, including medical devices, rapid prototyping, educational tools, and consumer products. The variations of PLA such as poly-L-lactic acid (PLLA), poly-D-lactic acid (PDLA), and poly-DL-lactic acid (PDLLA) are less frequently used in 3D printing due to their distinct physical characteristics compared to the standard PLA. PCL is a flexible biodegradable material and mechanically stable, making it suitable for biomedical applications such as tissue engineering and drug delivery systems [26]. PDO, another widely used biodegradable polymer in the medical field, offers a favorable balance of flexibility, tensile strength, and biodegradation time. Other important biodegradable polymers include polyhydroxyalkanoates (PHAs), a family of natural polyesters produced by bacterial fermentation of sugars or lipids. PHAs are highly biocompatible and are used in medical, agricultural, and packaging applications [27]. Thermoplastic starch (TPS) blends, derived from sources like corn or potatoes, are compostable and are often combined with other polymers to enhance their mechanical performance. Lignin-based filaments, a byproduct of the paper industry, provide good mechanical properties when blended and are increasingly used as a sustainable option in 3D printing [28]. Gelatin-based materials, derived from collagen, are biocompatible and widely applied in biomedical fields such as tissue engineering and regenerative medicine [29]. Algae-based filaments, made from algal biomass, offer a renewable and biodegradable alternative mainly used in sustainable design and prototyping [30]. These biodegradable materials are advancing in quality and variety, driven by the increasing demand for sustainable manufacturing practices. They offer a range of mechanical properties and biodegradability options, making them suitable for different applications from consumer products to medical devices.

The selection of PCL and PDO for the fabrication of 3D-printed tracheal stents is based on their well-established biocompatibility, biodegradability, and compatibility with FFF technology [25]. PCL is a widely studied biodegradable polyester characterized by a low melting point, excellent thermal stability, and ease of processing, which makes it particularly suitable for 3D printing of complex, patient-specific geometries. Its slow degradation rate also makes it attractive for applications requiring long-term structural support. PDO is commonly used in absorbable sutures and has demonstrated promising results in temporary implantable devices, including stents and scaffolds, due to its biocompatibility and intermediate degradation rate (typically 6 months) making it useful for short-term support [31]. Both polymers are commercially available in filament form and can be processed without the need for toxic solvents, contributing to safer and more sustainable manufacturing workflows. While other biodegradable polymers such as PLA and variations of PLA or PLGA are also available in filament form, their mechanical properties and degradation behavior are less optimal for airway stents: PLA tends to be brittle and degrades into acidic byproducts, while PLGA often resorbs too rapidly and may trigger local inflammation. Similarly, newer alternatives like TPS, gelatin-based, or algae-based filaments, while promising for sustainability or flexibility, currently lack extensive clinical validation and may pose challenges in reproducibility, mechanical stability, or biocompatibility in airway environments. In contrast, PCL and PDO provide a well-established balance of flexibility, thermal stability during printing, and biocompatibility, with a lower risk of adverse tissue reactions, key factors for safe and effective use in the dynamic tracheal environment. In addition, a PLA/PCL blend was included to explore the potential for tailoring mechanical and degradation properties by leveraging PLA’s rigidity and faster degradation with PCL’s ductility and processability. This combination provides greater design flexibility and may enhance the development of optimized, patient-specific bioresorbable airway stents.

In the present study, tracheal stents will be manufactured using 3D printing with an identical geometric design and three different biodegradable materials. Two key mechanical properties will be comparatively analyzed: flexural and radial force. These properties are critical for the functional behavior of the stent: flexural stiffness influences its ability to adapt to anatomical curvatures without causing mucosal injury [32,33,34], while radial force determines its capacity to keep the airway open without collapsing [22,35,36]. Differences in these properties depending on the material used could directly impact surgical planning and intraoperative execution [37].

Therefore, the main objective of this study is to evaluate and compare the mechanical properties, radial force, and bending of the same model of biodegradable tracheal stent manufactured using three different materials. The novelty of this work lies in its direct relationship between the specific mechanical behavior of each material and its 3D printing manufacturing process with the impact that they could have on surgical implantation practices. Through this approach, the study aims to demonstrate how differences in stiffness, elasticity, and expansion capacity between materials influence the placement procedure.

## 2. Materials and Experimental Methodology

**Figure 1:**
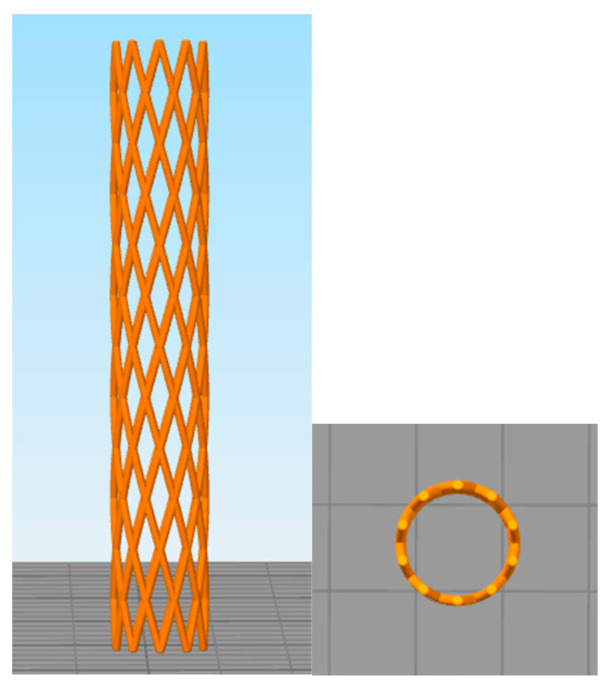
Three-dimensional image of the stent design with the X-pattern.

The stent design selected for this study follows a widely recognized X-pattern with a nominal diameter of 7 mm and a total length of 43 mm, inspired by commercially available metallic stents such as WallStent© (Boston Scientific, Marlborough, MA, USA). This type of configuration has been extensively reported on in the literature due to its ability to provide an effective balance, making it suitable for airway applications (Figure 1).

**Figure 2:**
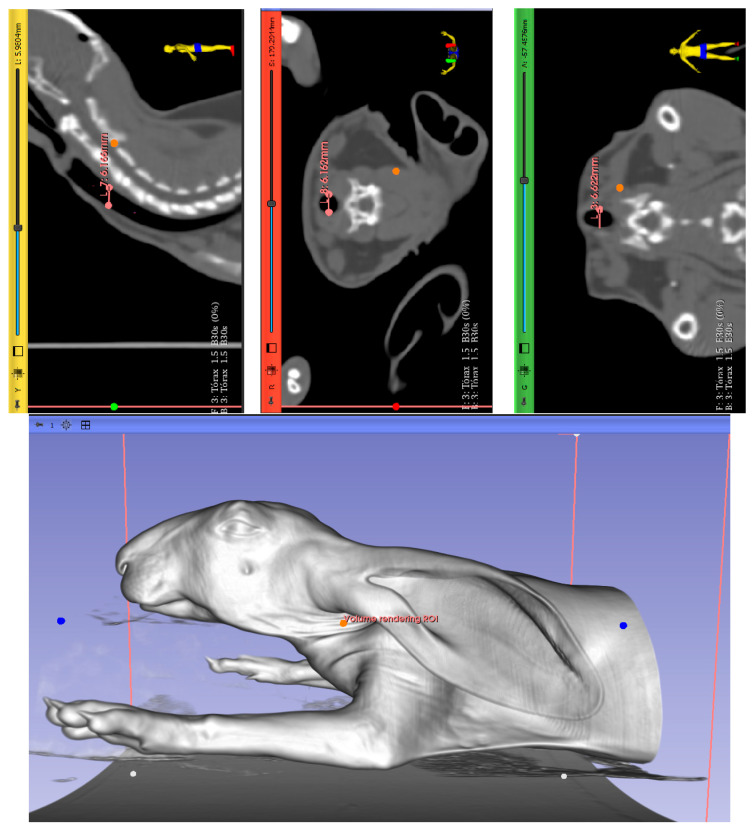
Ex vivo study: computerized tomography images of a cadaveric rabbit head, neck, and thorax. (**Upper left**): sagittal view. (**Upper center**): axial view. (**Upper right**): coronal view. (**Lower**): 3D view volume.

The initial diameter was selected based on a computerized tomography (CT) scan from a rabbit cadaver (Figure 2). The stents required adaptation to fit the dimensions of the rabbit trachea, which were measured with the open-source software 3DSlicer (The Slicer Community, Brigham and Women’s Hospital, Harvard Medical School, Boston, MA, USA, Version 5.6.2), revealing a diameter of 6.3, 6.1, and 6.6 mm. Measurements were obtained for the sagittal, axial, and coronal view and it is shown in the four images, marked with dots and with the dimension highlighted in orange.. In all cases, less than 7 mm was finally fixed as the initial dimension of the device in order to achieve optimal interaction between the device and the anatomical structure, and to provide a stent with minimal dimensions that ensure proper handling during the procedure.

The choice of this standardized and well-established geometry aims to eliminate the influence of structural variability in mechanical and performance outcomes. By maintaining a consistent design across all samples, the focus of this work remains exclusively on the comparative analysis of the different biodegradable materials used in the fabrication process. This approach ensures that the observed differences in mechanical behavior or printability are attributable to the intrinsic properties of each material rather than variations in stent geometry.

### 2.1. Selection of Materials

In this study, three biodegradable polymeric materials were selected based on their established mechanical performance and widespread use in biomedical applications: PCL, PDO, and a 50:50 polymeric blend of PLA and PCL (Table 1).

**Figure 3:**
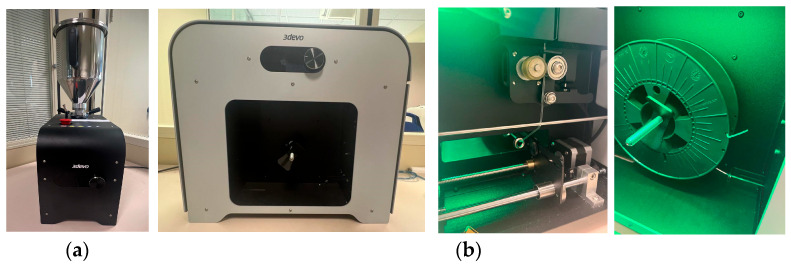
(**a**) Air Dryer used to dry PLA and PCL pellets. (**b**) Filament Maker-Composer used to manufacture the copolymer PLA/PCL filament.

The first two materials used in this study (PDO and PCL) were acquired directly from the supplier in filament form from Lattice Medical (Lattice Services, Loos, France), which were already pre-processed by the manufacturers and therefore did not require additional drying or extrusion steps prior to printing. In contrast, to obtain the PLA/PCL (50:50) polymeric blend, it was necessary to prepare the material in the laboratory. Starting from individual pellets of PLA obtained directly in pellet form from 3Devo (3Devo B.V., Utrecht, The Netherlands), we ground the PCL filament into pellets. The pellets of each polymer were dried separately prior to extrusion. Both materials were preconditioned through a drying process, with specific parameters optimized for each material (see Table 2), using the AIR DRYER from 3Devo (3Devo B.V., Utrecht, The Netherlands) (Figure 3). This step allowed the removal of residual moisture and improved the adhesion between polymers during extrusion, ensuring better quality of the final filament. After extrusion, the two dried polymers were combined in the desired ratio and co-fed into the twin-screw extruder to produce a homogeneous filament, using the filament extruder Filament Maker-Composer from 3Devo (3Devo B.V., Utrecht, The Netherlands) (Figure 3), obtaining a filament with a diameter of 1.75 mm under fixed parameters (see Table 3), suitable for use in FFF 3D printers.

### 2.2. Manufactured Methods

**Figure 4:**
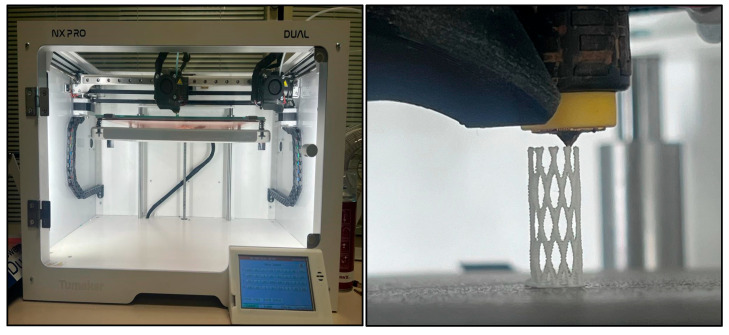
NX PRO Dual Filament–Filament printer and printing of the stent along the *Z*-axis on the 3D printer using 0.25 mm nozzle diameter.

The fabrication of the stents was carried out using FFF 3D printing technology with an NX PRO Dual Filament–Filament printer (Tumaker, Indart 3D, Irún, Spain) (Figure 4). This equipment features a dual extruder system and enables precise processing of multiple polymer types for complex geometries. The stent design in STL format was loaded into the software, where the printing orientation along the *Z*-axis was set to ensure correct layer deposition.

The printing process control and model preparation were performed using Simplify3D^®^ software, version 4.1.2 (Simplify3D LLC, Cincinnati, OH, USA), which allows advanced customization of printing parameters. For each of the materials used (PCL, PDO, and PLA–PCL 50:50), parameters were individually adjusted to optimize geometric fidelity to the base stent design and ensure the structural integrity of the printed model (see Table 4). These parameters were specifically optimized for each of the materials in order to ensure proper interlayer adhesion, dimensional accuracy, and structural quality of the complex geometries. Tailoring these settings is essential to guarantee the reproducibility and mechanical performance of the printed devices.

**Figure 5:**
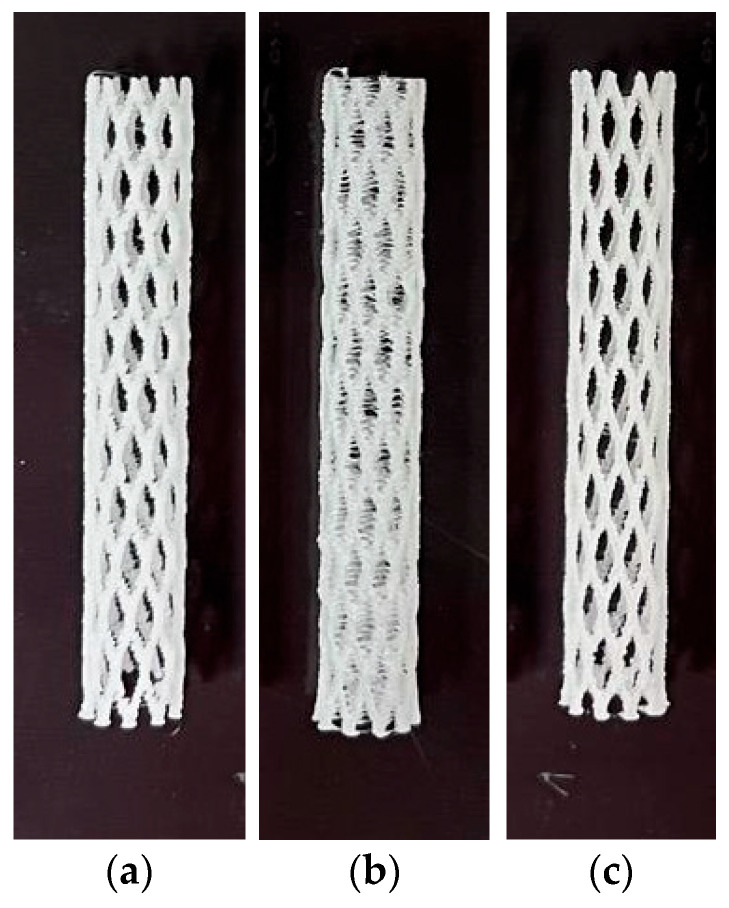
(**a**) Airway stent manufactured with PCL. (**b**) Airway stent manufactured with PDO. (**c**) Airway stent manufactured with the polymeric blend PLA/PCL (50:50).

The design parameters considered for these stents are based on a previous study conducted by Ayechu et al. [41], which aimed to evaluate the role of different geometric characteristics for manufacturing. Thus, the considered parameters are those shown in Table 5, and the stents resulting from 3D printing are shown in Figure 5.

### 2.3. Determination of Mechanical Properties

**Figure 6:**
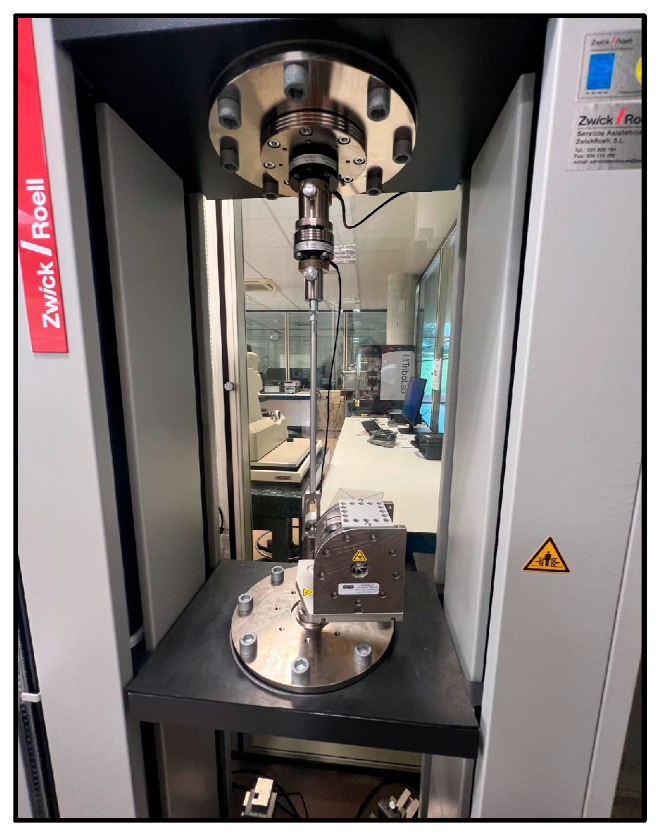
Zwick Roell universal testing machine with radial module.

Mechanical characterization of the 3D-printed stents was carried out using a Zwick Roell universal testing machine (Zwick GmbH & Co. KG, Ulm, Germany) equipped with specific modules (see Figure 6). This machine provides high precision in load and displacement control, which is essential for evaluating biomedical devices with small geometries and polymeric materials.

**Figure 7:**
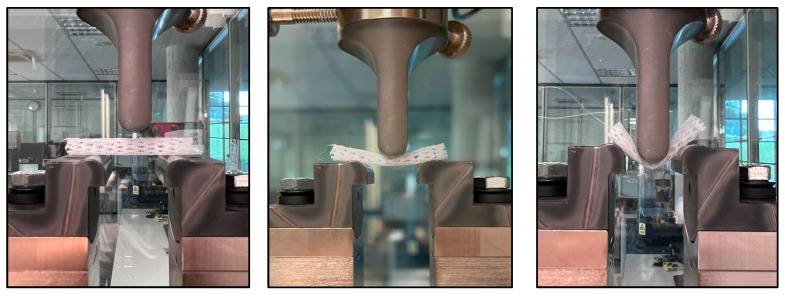
Three-point flexure test performed on one of the 3D-printed tracheal stents.

The flexure tests were conducted using a module with an adjustable support, suitable for small-sized specimens, and a calibrated load cell capable of recording forces within the low-deformation range. To evaluate the flexural behavior of the stent materials, a three-point bending test was performed. This test method, widely used for polymeric materials, involves placing the specimen horizontally on two supports and applying a vertical load at the midpoint until deformation or failure occurs (see Figure 7). The test was conducted following ISO 178 [42] standard specifications for determining flexural properties of plastics. A preload of 0.1 MPa was applied to ensure proper contact, with a flexural modulus test speed of 1 mm/min and a general test speed of 5 mm/min. The load and displacement data were recorded continuously to obtain the stress–strain curve, from which the flexural modulus, maximum flexural strength, and deformation at maximum flexural strength were calculated. This test allows for the comparison of stiffness and bending resistance across different materials used in the stent fabrication.

**Figure 8:**
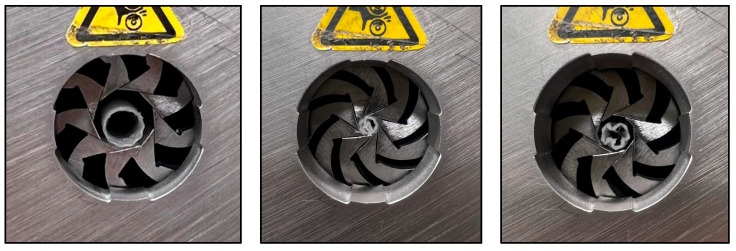
Radial force compression test performed on one of the 3D-printed tracheal stents.

To assess the radial mechanical performance of the manufactured stents, a radial compression test was conducted following a cyclic loading protocol based on ASTM F3067, which is commonly used to evaluate the radial strength of tubular medical devices (see Figure 8) [43]. The test consisted of radially compressing the stent until its external diameter was reduced by 3 mm, simulating the physiological loads experienced within the trachea. The compression was applied at a constant rate of 10 mm/min, while the force–displacement data were continuously recorded. The results provide critical information regarding the ability of the stent to maintain airway patency under physiological conditions without permanent deformation or collapse.

## 3. Results

The results shown in this section are calculated from three samples tested for each material. This experimental approach allowed for triplicate results for each test, thereby ensuring greater reliability, reproducibility, and the potential use of the data as a reference for future comparative studies.

### 3.1. Flexural Test

**Figure 9:**
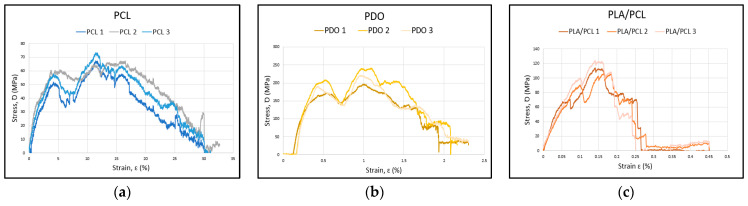
Curves from flexure test results: (**a**) PCL curve, (**b**) PDO curve, (**c**) PLA/PCL curve.

The experimental flexibility results for the three stent materials are shown in Table 6. The strain-stress curve corresponding to the three materials during the bending process is presented (Figure 9). This curve is essential for understanding the stiffness, ductility, and toughness of the materials. The numerical result numbers are presented in Table 6.

The results show that PLA/PCL is the material with the highest flexural modulus (E_f_) (4140 MPa, 3340 MPa, and 3138 MPa), indicating that it is the stiffest among the three materials. However, the strain at maximum flexural strength (ε_fM_) is the lowest among the materials studied (2.1%, 2.4%, and 3.5%), suggesting that it is a stiff but brittle material. On the other hand, PCL exhibits the lowest flexural modulus (1460 MPa, 1530 MPa, and 2020 MPa), making it the most flexible of the three. Moreover, the PCL samples show curves with low stress values (18.2 MPa, 17.6 MPa, and 19.1 MPa) but with a high deformation capacity before failure, highlighting its ductile behavior, similar to that observed in the PDO samples. The highest flexural strength is achieved by the samples manufactured with PDO (51.3 MPa, 63 MPa, and 57.7 MPa).

**Figure 10:**
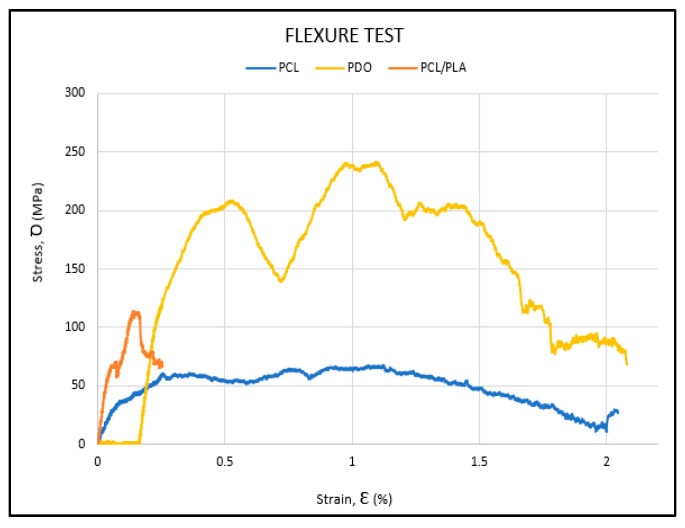
Representative curve from flexural test of each material.

To compare the bending curves of each material, the curve of a representative specimen from each is shown in the same graph (see Figure 10). PCL (blue curve) shows a more flexible and ductile behavior, with lower mechanical performance than PDO (yellow curve). In contrast, the PLA/PCL blend (orange curve) displays higher stiffness and flexural strength than PCL, though with reduced plasticity and a more brittle failure.

### 3.2. Radial Test

**Figure 11:**
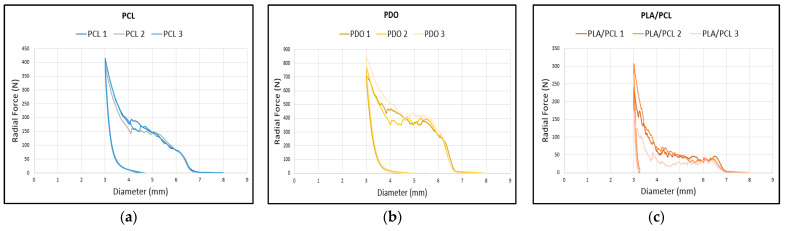
Curves from radial test results: (**a**) PCL curve, (**b**) PDO curve, (**c**) PLA/PCL curve.

The radial force test consists of applying a uniform compression along the stent’s axis to evaluate its resistance to collapse. In this study, a diameter reduction of 4 mm was established, compressing the stent from an initial diameter of 7 mm down to 3 mm, in order to get a similar compression which could suffer the stent during the implantation process in the surgical technique. The numeric results of the radial test are shown in Table 7, and the graphs of each material are shown in Figure 11. In the graphs, the *y*-axis represents the radial force in newtons (N), while the *x*-axis indicates the sample diameter in millimeters (mm).

The material that exhibits the highest radial compression force is PDO (773.18 N, 760.67 N, and 744.85 N). Among the other two samples, the PLA/PCL blend (531.1 N, 441.85 N, and 463.33 N) reaches higher values than the stents made of PCL (404.97 N, 396.22 N, and 377.26 N). This is expected, as the addition of PLA to the PCL polymer reinforces the material, increasing its stiffness and, consequently, enhancing its resistance to radial compression.

**Figure 12:**
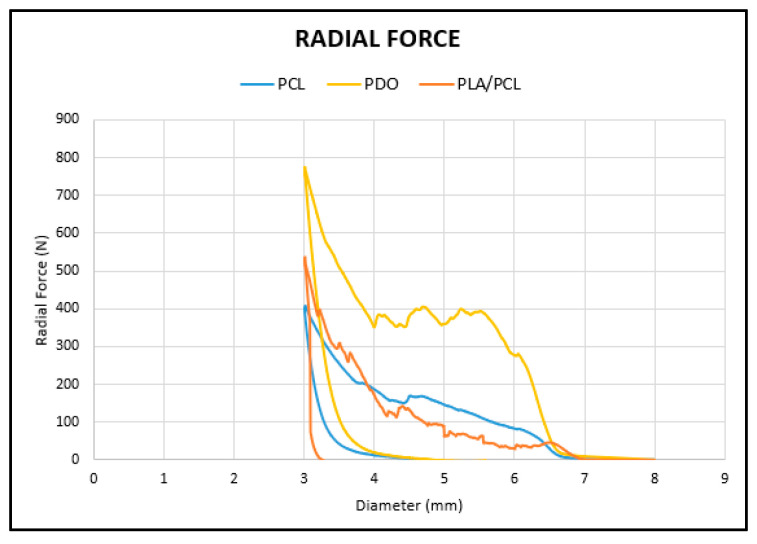
Representative curve of each material from radial test.

As in the case of the flexural test, to compare the radial curves of each material, the curve of a representative specimen from each is shown in the same graph (Figure 12).

PDO (yellow curve) exhibits the highest resistance to radial compression, while the PLA/PCL blend (orange curve) shows improved strength over pure PCL (blue curve) due to increased rigidity. However, the brittleness of PLA leads to complete fracture of the PLA/PCL stent upon unloading.

## 4. Discussion

Despite their widespread use in the management of airway disorders, tracheobronchial stents continue to present significant clinical limitations, often leading to controversial outcomes and a high rate of complications that necessitate further intervention [17]. Silicone stents are particularly prone to migration and blockage, while metallic ones, though generally thinner and more flexible, can provoke marked tissue reactions such as inflammation, granuloma development, and abnormal cell proliferation [7,9,44,45,46,47,48]. Procedurally, metallic stents offer the advantage of being placed under conscious sedation via flexible bronchoscopy, whereas silicone stents typically require general anesthesia and rigid bronchoscopy [4,9]. In contrast, biodegradable stents have emerged as a theoretically promising alternative, combining the structural benefits of metallic and silicone devices with the added advantage of gradual degradation, which could potentially reduce long-term complications. Preliminary trials conducted have shown limited success, often requiring intervention due to rapid degradation of the stent [49,50]. These outcomes highlight the need for further research before such devices can be considered viable substitutes for conventional stents [17,51]. These studies also underscore the importance of optimizing material composition to tailor degradation timelines to specific clinical scenarios.

This review of the scientific literature shows that more advanced knowledge is needed to design a biodegradable stent that overcomes current limitations. Therefore, in this work, we analyzed the mechanical properties, specifically radial force and flexural strength, to analyze the influence of the choice of material selection of 3D printable airway stents on their handling behavior, in order to try to predict their performance in terms of optimizing their use in clinical procedures [46].

The results obtained in this study are useful to know that the choice of material for tracheal stent manufacturing influences its mechanical properties, which could impact clinical handling and the implantation process. It was observed that materials with a higher elastic modulus, such as PDO, provide greater structural resistance, which could facilitate insertion without undesired deformations. However, this rigidity may also hinder anatomical adaptation to irregular or dynamic airways. In contrast, more flexible materials like PCL, although exhibiting lower radial compression resistance, offer greater deformability, which could ease placement in complex anatomies but compromise long-term stability. The PLA/PCL polymer blend showed intermediate behavior, combining rigidity and some flexibility, although its fragility at the end of the tests suggests a potential clinical limitation. These findings highlight the importance of selecting materials not only based on their biocompatibility and biodegradability, but also on their mechanical response, as this greatly determines the ease of handling and functional effectiveness of the stent in surgical settings.

These findings are partially consistent with previous studies that emphasize PLA’s rigidity and PCL’s flexibility as key characteristics in their biomedical application. For instance, research by She et al. [18] pointed out that materials with a higher elastic module provide better structural support but carry a greater risk of tracheal injury during insertion. Likewise, studies, such as those by McMahon et al. [52], show the importance of balancing mechanical strength and anatomical adaptability. In this context, our results further support the need to consider the individual mechanical properties of each material during surgical planning. Moreover, the work by She et al. [18] also reported that 3D-printed stents made from PLA exhibited high structural rigidity and sufficient radial force to maintain airway patency, but at the cost of limited flexibility, potentially increasing the risk of mucosal injury during placement. In contrast, PCL-based stents were shown to be more flexible and conformable, though with lower mechanical strength, which aligns with the trends observed in our study. Their study also emphasized the critical balance between mechanical robustness and anatomical adaptability, which aligns with the present work’s conclusion that material selection plays a central role in the clinical handling and long-term success of biodegradable stents.

### Limitations of the Study

Although the results obtained demonstrate how material selection influences the mechanical properties of tracheal stents, this study possesses certain limitations that should be taken into account. Firstly, the tests were conducted exclusively through experimental techniques under controlled laboratory conditions, which do not fully replicate the real clinical environment in which these devices are implanted. Moreover, the dynamic behavior of the stent during its insertion into the airway was not directly evaluated, an essential aspect for understanding its practical performance and ease of surgical handling. Future studies should address these aspects in order to validate the findings under real-life clinical conditions.

To overcome these limitations, future work will include the implementation of computational simulations aimed at modeling the movement of the stent during the insertion process. This approach would enable a more precise analysis of the forces exerted on the device within a virtual anatomical environment and allow comparison with the mechanical forces measured in laboratory tests using the universal testing machine. Once this comparison is made, it will be possible to establish a more accurate load profile for the stent, facilitating a correlation between its mechanical properties and its clinical applicability. This information will serve as a basis for the development of future in vivo trials, thus bringing the design of biodegradable stents closer to a more realistic and functional medical context.

## 5. Conclusions

This study highlights the importance of careful material selection in the design of 3D-printed tracheal stents, as it determines their mechanical properties and, consequently, their behavior during surgical manipulation. The ability to tailor these properties through polymer selection allows for the optimization of the balance between flexibility, strength, and ease of placement, which are key factors for improving clinical outcomes and reducing postoperative complications. These findings provide a solid foundation for the continued development of personalized stents adapted to the anatomical and therapeutic needs of each patient.

The results obtained in this study demonstrate that material selection has a decisive impact on the mechanical properties of 3D-printed tracheal stents, particularly in terms of flexural strength and radial force. These properties, in turn, could directly influence the stent’s behavior during surgical handling and the ease of device placement. PDO exhibited greater stiffness and strength, which could offer enhanced stability but less adaptability. In contrast, PCL showed higher flexibility, potentially facilitating insertion but with reduced structural support. The PLA/PCL (50:50) polymer displayed intermediate behavior, though its fragility represents a limitation. These findings underscore the need to match material choice to the specific requirements of the patient and lesion type, laying the groundwork for future development of clinically optimized and personalized stents.

## Figures and Tables

**Table 1:** Parameters of commercial materials PCL, PDO, and PLA [38,39,40].

	PCL	PDO	PLA Granules
Melting range (DSC, 10 °C/min)	50–70	95–110	150–180
Glass transition (DSC, 10 °C/min)	<−50	−21	55–60
Degradation temperature (°C)	>250	>240	>300
Molar mass (g/mol)	70,000–90,000	140,000–180,000	20,000–200,000
Density (g/cm^3^)	1.145	1.3	1.24
Chemical formula	C_6_H_10_O_2_	C_6_H_8_O_3_	C_3_H_4_O_2_

**Table 2:** Parameters established to dry the PLA and PCL pellets.

	PCL	PLA
Drying Temperature (°C)	40	60
Drying Time (°C)	5	47
Airflow Rate (%)	50	50

**Table 3:** Parameters established for each material to extrude the homogeneous filament.

		PLA/PCL Blend
Temperature Zones (°C)	Z1	160
	Z2	165
	Z3	140
	Z4	125
Screw Speed (rpm)		12
Fan Speed (%)		50
Filament Diameter (mm)		1.75

**Table 4:** Parameters established for each material to print the airway stent.

	PCL	PDO	PLA/PCL
Nozzle Temperature (°C)	160	150	190
Bed Temperature (°C)	45	45	45
Print Speed (mm/s)	20	20	40
Nozzle Diameter (mm)	0.25	0.25	0.25
Layer Height (mm)	0.2	0.2	0.2
Cooling Fan (%)	50	60	100
Retraction Distance (mm)	0.2	0.5	1
Retraction Speed (mm/s)	15	10	25
Bed Adherence	Texture Surface	Texture Surface	Texture Surface
Printing Time (min)	75 min	61 min	32 min

**Table 5:** Considered parameters for configurations of airway stents with X-patterns.

Parameters	X-Pattern
Pitch Angle α (°)	60
Wire Thickness *t* (mm)	0.25
Number of Peaks (or Cell Units) *p*	11
Diameter (mm)	7
Rings	12

**Table 6:** Flexural test results.

MATERIAL	E_f_ MPa	Ꝺ_fM_ MPa	ε_fM_ %
PCL 1	1460	18.2	15
PCL 2	1530	17.6	16
PCL 3	2020	19.1	11
PDO 1	2010	51.3	15
PDO 2	2900	63	16
PDO 3	3430	57.7	14
PLA/PCL 1	4140	29.7	2.1
PLA/PCL 2	3340	28	2.4
PLA/PCL 3	3138	32.5	3.5

**Table 7:** Radial test results.

MATERIAL	RFmax Apply N	RFmax Stand N/mm
PCL 1	404.97	9.18
PCL 2	396.22	9.21
PCL 3	377.26	8.77
PDO 1	773.18	17.98
PDO 2	760.67	17.69
PDO 3	744.85	17.32
PLA/PCL 1	531.10	12.35
PLA/PCL 2	441.85	10.27
PLA/PCL 3	463.33	10.77

## Data Availability

The original contributions presented in this study are included in the article. Further inquiries can be directed to the corresponding authors.

## References

[1] Rafanan A.L., Mehta A.C. (2000). Stenting of the Tracheobronchial Tree. Radiol. Clin. N. Am..

[2] Thornton R.H., Gordon R.L., Kerlan R.K., LaBerge J.M., Wilson M.W., Wolanske K.A., Gotway M.B., Hastings G.S., Golden J.A. (2006). Outcomes of Tracheobronchial Stent Placement for Benign Disease. Radiology.

[3] Delgado Pecellín I., González Valencia J.P., Machuca Contreras M., Pineda Mantecón M. (2009). Clinic, diagnosis and treatment of tracheal stenosis. An. Pediatr..

[4] Fruchter O., Raviv Y., Fox B.D., Kramer M.R. (2010). Removal of Metallic Tracheobronchial Stents in Lung Transplantation with Flexible Bronchoscopy. J. Cardiothorac. Surg..

[5] Bolliger C.T., Wyser C., Wu X., Hauser R., Studer W., Dalquen P., Perruchoud A.P. (1999). Evaluation of a New Self-Expandable Silicone Stent in an Experimental Tracheal Stenosis. Chest.

[6] Freitag L., Gördes M., Zarogoulidis P., Darwiche K., Franzen D., Funke F., Hohenforst-Schmidt W., Dutau H. (2017). Towards Individualized Tracheobronchial Stents: Technical, Practical and Legal Considerations. Respiration.

[7] Park H.Y., Kim H., Koh W.-J., Suh G.Y., Chung M.P., Kwon O.J. (2009). Natural Stent in the Management of Post-Intubation Tracheal Stenosis. Respirology.

[8] Sun F., Usón J., Ezquerra J., Crisóstomo V., Luis L., Maynar M. (2008). Endotracheal Stenting Therapy in Dogs with Tracheal Collapse. Vet. J..

[9] Dumon J.-F. (1990). A Dedicated Tracheobronchial Stent. CHEST.

[10] Saad C.P., Murthy S., Krizmanich G., Mehta A.C. (2003). Self-Expandable Metallic Airway Stents and Flexible Bronchoscopy*: Long-Term Outcomes Analysis. CHEST.

[11] Chung F.-T., Chen H.-C., Chou C.-L., Yu C.-T., Kuo C.-H., Kuo H.-P., Lin S.-M. (2011). An Outcome Analysis of Self-Expandable Metallic Stents in Central Airway Obstruction: A Cohort Study. J. Cardiothorac. Surg..

[12] Husain S.A., Finch D., Ahmed M., Morgan A., Hetzel M.R. (2007). Long-Term Follow-Up of Ultraflex Metallic Stents in Benign and Malignant Central Airway Obstruction. Ann. Thorac. Surg..

[13] Ríos A.E. (2006). Intervencionismo pulmonar: Broncoscopia rígida, cirugía endobronquial láser y prótesis traqueobronquiales. NCT Neumol. Cir. Tórax.

[14] Saito Y., Imamura H. (2005). Airway Stenting. Surg. Today.

[15] Gildea T.R., Young B.P., Machuzak M.S. (2018). Application of 3D Printing for Patient-Specific Silicone Stents: 1-Year Follow-Up on 2 Patients. Respir. Int. Rev. Thorac. Dis..

[16] Ghosh S., Burks A.C., Akulian J.A. (2019). Customizable Airway Stents-Personalized Medicine Reaches the Airways. J. Thorac. Dis..

[17] Guibert N., Saka H., Dutau H. (2020). Airway Stenting: Technological Advancements and Its Role in Interventional Pulmonology. Respirology.

[18] She Y., Fan Z., Wang L., Li Y., Sun W., Tang H., Zhang L., Wu L., Zheng H., Chen C. (2021). 3D Printed Biomimetic PCL Scaffold as Framework Interspersed with Collagen for Long Segment Tracheal Replacement. Front. Cell Dev. Biol..

[19] Guerra A.J., Cano P., Rabionet M., Puig T., Ciurana J. (2018). 3D-Printed PCL/PLA Composite Stents: Towards a New Solution to Cardiovascular Problems. Materials.

[20] Guerra A.J., Ciurana J. (2018). 3D-Printed Bioabsordable Polycaprolactone Stent: The Effect of Process Parameters on Its Physical Features. Mater. Des..

[21] Shen Y., Tang C., Sun B., Wu Y., Yu X., Cui J., Zhang M., El-Newehy M., El-Hamshary H., Barlis P. (2024). Development of 3D Printed Biodegradable, Entirely X-Ray Visible Stents for Rabbit Carotid Artery Implantation. Adv. Healthc. Mater..

[22] Thakur A., Vates U.K., Mishra S. (2024). Proof of Concept Study for Radial Compression Strength & Shape Memory Effect of 3D Printed Double Arrowhead PLA Stent. Emergent Mater..

[23] Sousa A.M., Amaro A.M., Piedade A.P. (2022). 3D Printing of Polymeric Bioresorbable Stents: A Strategy to Improve Both Cellular Compatibility and Mechanical Properties. Polymers.

[24] Zhao J., Song G., Zhao Q., Feng H., Wang Y., Anderson J.M., Zhao H., Liu Q. (2023). Development of Three-Dimensionally Printed Vascular Stents of Bioresorbable Poly(l-Lactide-Co-Caprolactone). J. Biomed. Mater. Res. B Appl. Biomater..

[25] Joseph T.M., Kallingal A., Suresh A.M., Mahapatra D.K., Hasanin M.S., Haponiuk J., Thomas S. (2023). 3D Printing of Polylactic Acid: Recent Advances and Opportunities. Int. J. Adv. Manuf. Technol..

[26] Malikmammadov E., Tanir T.E., Kiziltay A., Hasirci V., Hasirci N. (2018). PCL and PCL-Based Materials in Biomedical Applications. J. Biomater. Sci. Polym. Ed..

[27] Raza Z.A., Abid S., Banat I.M. (2018). Polyhydroxyalkanoates: Characteristics, Production, Recent Developments and Applications. Int. Biodeterior. Biodegrad..

[28] Jiang B., Jiao H., Guo X., Chen G., Guo J., Wu W., Jin Y., Cao G., Liang Z. (2023). Lignin-Based Materials for Additive Manufacturing: Chemistry, Processing, Structures, Properties, and Applications. Adv. Sci. Weinh. Baden-Wurtt. Ger..

[29] Jaipan P., Nguyen A., Narayan R.J. (2017). Gelatin-Based Hydrogels for Biomedical Applications. MRS Commun..

[30] Grira S., Khalifeh H.A., Alkhedher M., Ramadan M. (2023). 3D Printing Algae-Based Materials: Pathway towards 4D Bioprinting. Bioprinting.

[31] Athanasiou K.A., Niederauer G.G., Agrawal C.M. (1996). Sterilization, Toxicity, Biocompatibility and Clinical Applications of Polylactic Acid/Polyglycolic Acid Copolymers. Biomaterials.

[32] Bobel A.C., Petisco S., Sarasua J.R., Wang W., McHugh P.E. (2015). Computational Bench Testing to Evaluate the Short-Term Mechanical Performance of a Polymeric Stent. Cardiovasc. Eng. Technol..

[33] Chen C., Xiong Y., Li Z., Chen Y. (2020). Flexibility of Biodegradable Polymer Stents with Different Strut Geometries. Materials.

[34] Ferraro M., Auricchio F., Boatti E., Scalet G., Conti M., Morganti S., Reali A. (2015). An Efficient Finite Element Framework to Assess Flexibility Performances of SMA Self-Expandable Carotid Artery Stents. J. Funct. Biomater..

[35] McGrath D.J., Thiebes A.L., Cornelissen C.G., O’Brien B., Jockenhoevel S., Bruzzi M., McHugh P.E. (2018). Evaluating the Interaction of a Tracheobronchial Stent in an Ovine In-Vivo Model. Biomech. Model. Mechanobiol..

[36] McGrath D.J., Thiebes A.L., Cornelissen C.G., O’Shea M.B., O’Brien B., Jockenhoevel S., Bruzzi M., McHugh P.E. (2017). An Ovine in Vivo Framework for Tracheobronchial Stent Analysis. Biomech. Model. Mechanobiol..

[37] Venkatraman S.S., Tan L.P., Joso J.F.D., Boey Y.C.F., Wang X. (2006). Biodegradable Stents with Elastic Memory. Biomaterials.

[38] PDO-Fiche-Technique-EN.Pdf. https://lattice-services.com/wp-content/uploads/PDO-Fiche-technique-EN.pdf.

[39] Lattice Medical, PCL-Fiche-Technique-EN.Pdf. https://lattice-services.com/wp-content/uploads/PCL-Fiche-technique-FR.pdf.

[40] NatureWorks LLC Technical Data Sheet 4043D 3D Monofilament, [Online]. https://www.natureworksllc.com/~/media/Files/NatureWorks/Technical-Documents/Technical-Data-Sheets/TechnicalDataSheet_4043D_3D-monofilament_pdf..

[41] Ayechu-Abendaño A., Pérez-Jiménez A., Sánchez-Matás C., López-Villalobos J.L., Díaz-Jiménez C., Fernández-Parra R., Malvè M. (2024). Computational Analysis of Polymeric Biodegradable and Customizable Airway Stent Designs. Polymers.

[42] (2019). Plastics—Determination of Flexural Properties.

[43] Ensayos en Stents. https://www.zwickroell.com/es/sectores/industria-medica-y-farmaceutica/cateteres-y-stents/ensayos-en-stents/.

[44] Noppen M. (2025). Airway Injury and Sequelae: Conservative View. Surgery for Non-Neoplastic Disorders of the Chest: A Clinical Update.

[45] Melgoza E.L., Serenó L., Rosell A., Ciurana J. (2012). An Integrated Parameterized Tool for Designing a Customized Tracheal Stent. Comput.-Aided Des..

[46] Xavier R.G., Sanches P.R.S., de Macedo Neto A.V., Kuhl G., Vearick S.B., Michelon M.D. (2008). Development of a modified Dumon stent for tracheal applications: An experimental study in dogs. J. Bras. Pneumol..

[47] Zurita-Gabasa J., Sánchez-Matás C., Díaz-Jiménez C., López-Villalobos J.L., Malvè M. (2021). A Parametric Tool for Studying a New Tracheobronchial Silicone Stent Prototype: Toward a Customized 3D Printable Prosthesis. Mathematics.

[48] Malvè M., Barreras I., López-Villalobos J.L., Ginel A., Doblaré M. (2012). Computational Fluid-Dynamics Optimization of a Human Tracheal Endoprosthesis. Int. Commun. Heat Mass Transf..

[49] Lischke R., Pozniak J., Vondrys D., Elliott M.J. (2011). Novel Biodegradable Stents in the Treatment of Bronchial Stenosis after Lung Transplantation. Eur. J. Cardio-Thorac. Surg..

[50] Zając A., Krysta M., Kiszka A., Górecki W. (2019). Biodegradable Airway Stents: Novel Treatment of Airway Obstruction in Children. Adv. Clin. Exp. Med..

[51] Dutau H., Reynaud-Gaubert M., Thomas P.A. (2012). Endoscopic Management of Post-Lung Transplantation Anastomotic Stenosis: Metallic, Silicone or Biodegradable Stents. Eur. J. Cardio-Thorac. Surg..

[52] McMahon S., Bertollo N., O’Cearbhaill E.D., Salber J., Pierucci L., Duffy P., Dürig T., Bi V., Wang W. (2024). Bio-Resorbable Polymer Stents: A Review of Material Progress and Prospects. Prog. Polym. Sci..

